# Clinical characteristics of type 2 diabetes mellitus patients with erectile dysfunction

**DOI:** 10.3389/fendo.2026.1780498

**Published:** 2026-03-04

**Authors:** Jiping Cao, Yang Zhang, Wei Jiang, Ying Fang, Yele Zhang, Lin Jiang

**Affiliations:** 1 Department of Nutrition, Jiangsu Provincial People’s Hospital, First Affiliated Hospital of Nanjing Medical University, Nanjing, China; 2 Department of Radiology, Jiangsu Provincial People’s Hospital, First Affiliated Hospital of Nanjing Medical University, Nanjing, China

**Keywords:** anxiety, clinical features, depression, erectile dysfunction, type 2 diabetes mellitus

## Abstract

**Background:**

Type 2 diabetes mellitus (T2DM) is a prevalent metabolic disorder and erectile dysfunction (ED) is a common complication among male patients with T2DM. Although T2DM is a well-established independent risk factor for ED the clinical characteristics and associated psychosocial burden of ED in this population remain inadequately characterized. This study aimed to comprehensively assess the prevalence, severity, clinical correlates, and emotional comorbidities of ED in male patients with T2DM.

**Objective:**

The incidence and related factors of T2DM patients with ED (T2DMED) were investigated by clinical symptom collection and related scale evaluation. The emotional abnormalities of T2DMED patients and their relationships with clinical features were evaluated by the Hamilton Anxiety Scale (HAMA) and Hamilton Depression Scale (HAMD).

**Methods:**

A total of 208 male patients with T2DM who presented to the Department of Endocrinology in Jiangsu Provincial People’s Hospital from July 2020 to March 2021 were selected. The demographic information and T2DM related clinical data were collected. The scales of HAMA, HAMD, and sexual function questionnaires including International Erectile Function Index (IIEF-5), Premature Ejaculation Diagnostic Tool (PEDT) and Arizona Sexual Experience Scale (ASEX) were evaluated. Statistical analysis was conducted based on the patient’s demographic information, clinical indicators, and scale data.

**Results:**

The prevalence of ED in male patients with T2DM was 67.8%. The prevalences of mild, moderate and severe ED were 57.2%, 5.3%, 5.3%, respectively. Compared with the group of T2DM, higher age, lower IIEF score, higher PEDT score, higher ASEX score, increased left and right pulse wave velocity (PWV) were detected in the T2DMED group. In addition, age and fasting insulin had positive impacts on the development of ED in T2DM patients. HbA1C, age and educational level had impacts on the severity of ED. The severity of ED was positively correlated with low-density lipoprotein (LDL) level, age, left ankle brachial index (ABI), left and right PWV. There were negative correlations between IIEF-5 scores and HAMA, HAMD scores in T2DM patients. PEDT scores were positively correlated with HAMA and HAMD scores while ASEX scores were positively correlated with HAMA scores. The HAMA scores were positively correlated with the duration of T2DM and HAMA scores had negative effects on IIEF scores.

**Conclusion:**

Mild ED is more common in T2DM patients with more serious sexual dysfunction and higher risk of diabetic vasculopathy. Higher age and fasting insulin level are associated with the development of T2DMED while HbA1C, age and education level affect the severity of ED, with higher levels of low-density lipoprotein (LDL) and age, high risk of diabetic vasculopathy indicating more severe ED. The sexual dysfunction was positively associated with both anxiety and depression, especially in patients with higher age.

## 1 Introduction

Type 2 diabetes mellitus (T2DM) is a prevalent metabolic disorder characterized by insulin resistance and insufficient insulin secretion, leading to chronic hyperglycemia (1). Global projections indicate that the number of individuals with diabetes will rise to approximately 300 million by 2025 and 693 million by 2045 (2). T2DM is frequently accompanied by a spectrum of complications that adversely affect multiple organ systems, including the cardiovascular system, eyes, kidneys, endocrine glands, and peripheral nerves (3–7). Moreover, T2DM is associated with an increased risk of cognitive impairment and emotional disorders (4–8). These complications contribute to elevated healthcare costs, reduced quality of life, and increased mortality (9). Consequently, the prevention and management of T2DM-related complications warrant significant clinical attention.

Erectile dysfunction (ED) is defined as the persistent inability to attain or maintain a penile erection sufficient for satisfactory sexual performance (10). Current evidence indicates that ED affects over 150 million men globally, with projections suggesting this figure may reach 322 million by 2025 (11). Multiple etiological factors contribute to ED, including vascular diseases, obesity, smoking, metabolic syndrome, hyperlipidemia, depression, medication side effects, diabetes mellitus, and neurological disorders (12, 13). Among these, T2DM is recognized as an independent risk factor for ED, with pathogenesis involving several interrelated mechanisms (14). The condition is commonly diagnosed using the widely adopted International Index of Erectile Function-5 (IIEF-5) questionnaire (15). According to the IIEF-5 scoring system, a score ≤21 indicates the presence of ED, with further stratification into mild (12–21), moderate (8–11), and severe (0–7) categories, while a score of 22–25 suggests normal erectile function (16, 17). ED substantially impairs men’s quality of life and self-esteem (18) and poses a considerable economic burden on healthcare systems.

Beyond its status as an independent risk factor, the association between T2DM and ED is underpinned by a complex, multifactorial pathophysiology that merits further investigation (19, 20). Chronic hyperglycemia, a hallmark of T2DM, initiates a cascade of deleterious processes (21). It promotes the formation and accumulation of advanced glycation end products (AGEs) in vascular and neural tissues, leading to endothelial dysfunction (22) and reduced bioavailability of nitric oxide (NO)—a key mediator of penile vasodilation (23). Concurrently, hyperglycemia-induced oxidative stress and chronic low-grade inflammation exacerbate endothelial and neural damage (24). Insulin resistance, another central feature of T2DM, further aggravates this process by promoting both macrovascular and microvascular complications, such as atherosclerosis and microangiopathy, which impede blood flow to the corpora cavernosa (25). Additionally, diabetic autonomic neuropathy can disrupt the intricate neural signaling essential for initiating and sustaining an erection (26). Together, these vascular, neurological, and endothelial impairments create a pathophysiological ‘perfect storm’ that fosters ED in T2DM patients, positioning ED not merely as a complication but as a potential sentinel marker of underlying systemic vasculopathy (27).

The influence of psychological comorbidities—particularly anxiety and depression—on the development and progression of ED in individuals with diabetes represents a critical yet frequently overlooked dimension in clinical management (28). T2DM itself constitutes a chronic stressor, imposing a significant psychological burden related to daily self-management demands, fears of complications, and lifestyle adjustments (29). Such psychological distress may directly contribute to ED via neuroendocrine pathways, including chronic activation of the hypothalamic-pituitary-adrenal (HPA) axis and sympathetic nervous system, resulting in elevated cortisol and catecholamine levels (30). These hormones can induce peripheral vasoconstriction, inhibit NO synthesis, and diminish sexual desire (31). Conversely, the onset of ED can be psychologically distressing, leading to performance anxiety, diminished self-esteem, interpersonal strain, and social withdrawal, which may further compromise glycemic control through poor treatment adherence and maladaptive coping behaviors (32). This interaction establishes a vicious, self-perpetuating cycle wherein suboptimal metabolic health exacerbates ED, ED heightens psychological distress, and psychological distress, in turn, worsens metabolic control (33). Elucidating this bidirectional relationship is therefore essential for developing integrated, patient-centered interventions aimed at breaking this cycle.

Given the substantial clinical and psychosocial impact of ED in T2DM, focused attention on this patient subgroup is imperative. In particular, systematic screening using instruments such as the IIEF-5 questionnaire, even before overt symptoms emerge, is of great importance (34). Early detection of ED and prompt intervention targeting modifiable risk factors can significantly alleviate both individual suffering and the broader societal burden (35). Accordingly, this study was designed as a cross-sectional, single-center investigation to evaluate the prevalence, associated risk factors, and clinical correlates of ED in male patients with T2DM.

## 2 Materials and methods

### 2.1 Participants

This study was approved by the Ethical Commission of Jiangsu Provincial People’s Hospital. All patients signed informed consents before participation. Male patients with T2DM who presented to the Department of Endocrinology in Jiangsu Provincial People’s Hospital from July 2020 to March 2021 were selected.

Diagnosis of T2DM: According to the diagnostic criteria of the American diabetes Association (ADA) on diabetes in 2018, it was believed that the fasting blood glucose was>7mmol/L, or the blood glucose in 2 hours after oral administration of 75g of anhydrous glucose dissolved in water was≥11.1mmol/L, or the glycosylated hemoglobin was≥6.5%, or patients with typical hyperglycemia or hyperglycemia crisis symptoms had random blood glucose≥11.1mmol/L (36).

Diagnosis of ED: According to the definition of the National Institutes of Health (NIH) in 1993, ED referred to the sustained inability to achieve or maintain an erection sufficient for sexual intercourse. At present, ED could be diagnosed based on the most widely used IIEF-5 questionnaire (15). According to the IIEF questionnaire scores, the severity of ED was graded, with scores ≤ 21 indicating the presence of ED (16), 22–25 indicating normal erectile function, scores between 12–21 indicating mild, 8–11 indicating moderate, and 0–7 indicating severe (17).

Inclusion criteria: (1) Diagnosis of T2DM; (2) Han ethnicity; (3) Right-handed; (4) Age ranged from 20 to 60 years old, this upper age limit was chosen to minimize the potential confounding effect of age-related non-diabetic vascular and neurological degenerative processes on erectile function, thereby allowing a clearer focus on the contribution of T2DM-specific factors; (5) Fixed sexual partner, regular sexual activity for more than 6 months.

Exclusion criteria: (1) Diagnosis of Type 1 diabetes, maturity-onset diabetes of the young, or other specific types of diabetes; (2) Sexual partner with sexual dysfunction; (3) Abnormal external genitalia; (4) Accompanied by severe physical or mental illnesses that would impede questionnaire completion or confound results; (5) History of radical pelvic surgery or pelvic radiotherapy; (6) Known primary hypogonadism or untreated severe hypogonadism; (7) Severe renal failure; (8) History of major neurological diseases known to affect sexual function; (9) Active malignancy or history of cancer treatment; (10) Drug abuse or use of drugs that affected sexual function.

### 2.2 Demographic and clinical information collection

Demographic data were collected, including gender, age, height, weight, body mass index (BMI), where BMI was calculated by dividing weight (kg) by the square of height (meters). In addition, the following measures were collected, including smoking history (smoking or non-smoking), drinking history (drinking or not drinking), educational level (illiteracy, primary school, junior high school, high school, vocational school, college, university, graduate school), HbA1C (HbA1C value within 3 months), T2DM duration (calculated by age of onset), history of hypertension (with or without hypertension), triglycerides (TG), total cholesterol (TC), low-density lipoprotein (LDL), urinary microalbumin (umALB), blood creatinine (Cr), blood urea nitrogen (Urea), fasting blood glucose (FBG), visceral fat area (VAT), ankle brachial index (ABI), pulse wave velocity (PWV), fasting insulin (Fins) levels, fasting C-peptide (FCP) levels.

### 2.3 Scales assessment

All patients completed the IIEF-5 (17), Premature Ejaculation Diagnostic Tool (PEDT) (37), Arizona Sexual Experience (ASEX) scales (38), Hamilton Anxiety Scale (HAMA) (39) and Hamilton Depression Scale (HAMD) (40, 41) scales.

### 2.4 Statistical analysis

The data was statistically analyzed using SPSS (version 23.0; IBM Corp., Armonk, NY, USA) software. Kolmogorov Smirnov was used to test whether each indicator conformed to a normal distribution. Metric data that conformed to normality were represented by Mean ± standard deviation, and *F* or *t* tests was used. Quantitative data that did not follow a normal distribution were represented by the median and *Mann-Whitney* rank sum test was used. Count data was expressed as percentages using *Pearson* chi square test or *Fisher’s* exact probability test. *Pearson* correlation, *Spearman* rank correlation, or *Partial* correlation were used for correlation analysis. The possible influencing factors of observation indicators were analyzed using binary logistic regression or ordered logistic regression.

## 3 Results

### 3.1 The probability of ED occurring in T2DM patients

A total of 208 male T2DM patients aged 20–60 years were collected, including 141 with ED (IIEF-5 score ≤ 21) and 67 without ED (IIEF-5 score>21). The prevalence of ED was 67.8% (141/208). Among them, there were 119 cases of mild ED (IIEF-5 score between 12-21), with a prevalence rate of 57.2% (119/208). There were 11 cases of moderate ED (IIEF-5 score 8-11), with a prevalence rate of 5.3% (11/208). There were 11 cases of severe ED (IIEF-5 score 0-7), with a prevalence rate of 5.3% (11/208) (**Table 1**).

**Table 1:** The probability of ED occurring in T2DM patients.

Severity of ED	Cases	Prevalence (%)
ED	141	67.3% (141/208)
Mild ED	119	57.2% (119/208)
Moderate ED	11	5.3% (11/208)
Severe ED	11	5.3% (11/208)

T2DM, type 2 diabetes mellitus; ED, erectile dysfunction.

### 3.2 Comparison of demographic and clinical data between T2DM and T2DMED groups

Compared with T2DM patients, T2DMED patients showed no significant differences in disease duration, height, weight, BMI, HbA1C, umALB, FBG, Fins, FCP, TC, TG, LDL, Cr, Urea, right ABI, left ABI, VAT, hypertension, smoking history, alcohol consumption history, and educational level (*P*>0.05). Compared with the T2DM group, the T2DMED group showed higher age (*P* = 0.004), lower IIEF-5 scores (*P* < 0.001), higher PEDT scores (*P* < 0.001), higher ASEX scores (*P* < 0.001), increased left and right PWV (*P* = 0.001) (**Table 2**).

**Table 2:** Comparison of demographic and clinical data between T2DMED and T2DM groups.

Characteristics	T2DMED	T2DM	*P*
	Mean ± standard deviation/Sum of ranks	Mean ± standard deviation/Sum of ranks	
Course of disease (years)	3	2	0.392
Age (years)	45.75 ± 7.38	41.22 ± 7.71	0.004
Height (cm)	170	173	0.076
Weight (kg)	74.84 ± 11.13	79.07 ± 13.64	0.096
BMI (kg/m2)	25.62 ± 3.22	26.27 ± 3.59	0.353
IIEF-5 (scores)	18	23	0.000
PEDT (scores)	6	3	0.000
ASEX (scores)	15	11	0.000
HbA1C (%)	8.52 ± 2.18	7.80 ± 2.19	0.172
umALB (mgL)	10.8	8.2	0.058
FBG (mmol/L)	6.87	6.48	0.62
Fins (pmol/L)	41.7	43.2	0.856
FCP (pmol/L)	603.9	626.6	0.5
TC (mmol/L)	4.72	4.9	0.666
TG (mmol/L)	1.33	1.87	0.079
LDL (mmol/L)	3.12 ± 0.96	2.89 ± 0.75	0.204
Cr (umol/L)	70.4 ± 10.7	71.83 ± 13.23	0.556
Urea (mmol/L)	5.7 ± 1.27	5.46 ± 1.15	0.359
Right ABI	1.09 ± 0.1	1.09 ± 0.07	0.866
Left ABI	1.07	1.09	0.407
Right PWV	1464 ± 246.25	1329 ± 139.85	0.001
Left PWV	1477.94 ± 222.54	1348 ± 144.22	0.001
VAT (cm2)	88.88 ± 33.26	93.57 ± 42.88	0.543
Hypertension (n, %)	25 (39.7)	15 (40.5)	0.933
Smoking (n, %)	30 (47.6)	21 (56.8)	0.377
Drinking (n, %)	30 (47.6)	21 (56.8)	0.377
Educational level			0.872
Middle school and below group (n, %)	11 (17.5)	5 (13.5)	
High school and vocational school group (n, %)	10 (15.9)	6 (16.2)	
College and above group (n, %)	42 (66.7)	26 (70.3)	

T2DM, type 2 diabetes mellitus; T2DMED, type 2 diabetes mellitus with erectile dysfunction; BMI, body mass index; IIEF-5, International Index of Erectile Function-5; PEDT, Premature Ejaculation Diagnostic Tool; ASEX, Arizona Sexual Experience Scale; HbA1c, glycated hemoglobin; umALB, urinary microalbumin; FBG, fasting blood glucose; Fins, fasting insulin; FCP, fasting C-peptide; TC, total cholesterol; TG, triglycerides; LDL, low-density lipoprotein; Cr, creatinine; ABI, ankle brachial index; PWV, pulse wave velocity; VAT, visceral fat area.

### 3.3 Binary logistic regression of factors influencing ED

The results showed that age had a positive effect on the development of ED in T2DM patients (*P* = 0.035), indicating that age was a risk factor for ED (OR value of 1.122). Older patients were 1.122 times more likely to develop ED compared to younger patients, which suggested that older T2DM patients were more likely to develop ED. Meanwhile, Fins had a positive impact on the development of ED in T2DM patients (*P* = 0.031), indicating that Fins was a risk factor for ED (OR value of 1.029). Patients with high Fins were 1.029 times more likely to develop ED compared to those with low Fins, suggesting that T2DM patients with high Fins were more likely to develop ED (**Table 3**).

**Table 3:** Binary logistic regression of factors influencing ED.

Variables	B	Standard error	*P*	Exp(B)	95%CI
Constant	42.439	81.127	0.601	2.699E+18	
Junior high school and below group	0.357	0.848	0.674	1.428	7.526
High school and vocational school group	-1.419	0.855	0.097	0.242	1.293
College and above group					
BMI	-0.783	1.543	0.612	0.457	9.396
Hypertension	-0.095	0.624	0.879	0.909	3.088
Drinking	-0.092	0.677	0.892	0.912	3.439
Smoking	0.780	0.612	0.203	2.181	7.244
Cr	0.016	0.03	0.592	1.016	1.078
Urea	0.405	0.257	0.115	1.500	2.483
LDL	0.343	0.531	0.518	1.409	3.991
TG	-0.018	0.201	0.927	0.982	1.456
TC	-0.035	0.412	0.933	0.966	2.164
HbA1C	0.247	0.184	0.179	1.281	1.838
umALB	0.002	0.005	0.690	1.002	1.012
Weight	0.281	0.515	0.585	1.325	3.638
Hight	-0.348	0.477	0.466	0.706	1.798
Course of disease	-0.02	0.094	0.835	0.98	1.18
Age	0.115	0.055	0.035	1.122	1.248
FBG	-0.126	0.107	0.236	0.881	1.086
Fins	0.028	0.013	0.031	1.029	1.056
FCP	-0.004	0.002	0.06	0.996	1.000
Left ABI	0.170	0.782	0.827	1.186	5.494
Right ABI	1.118	3.194	0.726	3.06	1602.254
Right PWV	0.000	0.003	0.878	1	1.006
Left PWV	0.004	0.004	0.222	1.004	1.011
VAT	0.002	0.011	0.875	1.002	1.023

ED, erectile dysfunction; BMI, body mass index; Cr, creatinine; LDL, low-density lipoprotein; TG, triglycerides; TC, total cholesterol; HbA1c, glycated hemoglobin; umALB, urinary microalbumin; FBG, fasting blood glucose; Fins, fasting insulin; FCP, fasting C-peptide; ABI, ankle brachial index; PWV, pulse wave velocity; VAT, visceral fat area; CI, confidence interval.

### 3.4 Ordered logistic regression analysis of ED severity

Based on the severity of ED as the dependent variable, it was divided into four levels: no ED, mild ED, moderate ED, and severe ED. The independent variables were HbA1C, BMI, disease duration, age, FBG, smoking, educational level, alcohol consumption, and hypertension. The results suggested that HbA1C, age, high school and vocational education were all risk factors that affected the severity of ED. The ordered logistic regression analysis suggested that HbA1c, age, and education level were significant factors. It should be noted that the distribution of ED severity was imbalanced, with mild ED being most prevalent (**Table 4**).

**Table 4:** Ordered logistic regression analysis of ED severity.

Variables	Standard error	Wald	*P*	95%CI
HbA1C	0.077	5.769	0.016	0.034, 0.377
BMI	0.055	2.946	0.086	-0.021, 0.013
Course of disease	0.036	0.259	0.611	-0.09, 0.053
Age	0.028	20.036	0.000	0.07, 0.179
FBG	0.059	0.009	0.924	-0.110, 0.122
No smoking	0.378	1.817	0.178	-0.232, 1.251
Smoking				
Junior high school and below group	0.466	1.592	0.207	-1.502, 0.326
High school and vocational school group	0.498	6.4	0.011	-2.234, -0.284
College and above group				
No drinking	0.377	0.963	0.326	-0.369, 1.11
Drink				
No hypertension	0.343	2.442	0.118	-1.209, 0.136
Hypertension				

ED, erectile dysfunction; HbA1c, glycated hemoglobin; BMI, body mass index; FBG, fasting blood glucose; CI, confidence interval.

### 3.5 Spearman correlation analysis between ED and demographic, clinical indicators

The ED grading was positively correlated with LDL levels (*r* = 0.256), age (*r* = 0.316), left ABI (*r* = 0.32), left PWV (*r* = 0.258), and right PWV (*r* = 0.011) (all *P* < 0.05) (**Table 5**).

**Table 5:** Spearman correlation analysis between ED and demographic, clinical indicators.

Variables	Smoking	Drinking	Hypertension	Education	Cr	Urea	LDL
ED severity (*r*, *P*)	0.059, 0.563	-0.094, 0.35	0.029, 0.777	0.001, 0.994	-0.008, 0.937	0.006, 0.955	0.256, 0.01
ED severity (*r*, *P*)	TG -0.131, 0.193	TC -0.114, 0.26	HbA1C 0.124, 0.219	umALB 0.046, 0.648	Weight 0.152, 0.132	Height -0.077, 0.449	
ED severity (*r*, *P*)	BMI -0.139, 0.169	Course of disease 0.186, 0.064	Age 0.316, 0.001	FBG 0.009, 0.93	Fins -0.001, 0.994		
ED severity (*r*, *P*)	Fasting C-peptide -0.116, 0.249	Left ABI 0.32, 0.001	Right ABI -0.1, 0.32	Right PWV 0.254, 0.011	Left PWV 0.258, 0.01	VAT -0.075, 0.456	

ED, erectile dysfunction; Cr, creatinine; LDL, low-density lipoprotein; TG, triglycerides; TC, total cholesterol; HbA1c, glycated hemoglobin; umALB, urinary microalbumin; BMI, body mass index; FBG, fasting blood glucose; Fins, fasting insulin; ABI, ankle brachial index; PWV, pulse wave velocity; VAT, visceral fat area.

The results suggested that HbA1c, age, and high school/vocational education level were significant risk factors affecting ED severity in the multivariable model. It is noteworthy that while the bivariate Spearman correlation between HbA1c and ED severity was not statistically significant, HbA1c became a significant predictor in the ordered logistic regression analysis after adjusting for other covariates. This indicates that the relationship between glycemic control and ED severity is more clearly discernible when accounting for potential confounders such as age, BMI, and hypertension status.

### 3.6 Binary logistic analysis of HAMA and HAMD scores on ED

The results showed that HAMA scores and HAMD scores were not influencing factors for whether T2DM patients had ED (**Table 6**).

**Table 6:** Binary logistic analysis of HAMA and HAMD scores on ED.

Variables	B	Standard error	*P*	EXP(B)	95%CI
Constant	-1.52	0.62	0.014	0.219	
HAMA scores	0.186	0.138	0.178	1.205	0.919, 1.58
HAMD scores	-0.007	0.159	0.964	0.993	0.727, 1.356

ED, erectile dysfunction; HAMA, Hamilton Anxiety Scale; HAMD, Hamilton Depression Scale; CI, confidence interval.

### 3.7 Spearman correlation analysis between ED and emotion

The results showed negative correlations between IIEF-5 scores and HAMA scores, as well as HAMD scores. There were positive correlations between PEDT scores and HAMA scores, HAMD scores. The ASEX scores were positively correlated with the HAMA scores (**Table 7**).

**Table 7:** Spearman correlation analysis between ED and emotion.

Variables	HAMA scores (*r*, *P*)	HAMD scores (*r*, *P*)
IIEF scores	(-0.403, 0.013)	(-0.329, 0.047)
PEDT scores	(0.386, 0.018)	(0.374, 0.022)
ASEX scores	(0.397, 0.015)	(0.19, 0.261)

ED, erectile dysfunction; HAMA, Hamilton Anxiety Scale; HAMD, Hamilton Depression Scale; IIEF, International Index of Erectile Function; PEDT, Premature Ejaculation Diagnostic Tool; ASEX, Arizona Sexual Experience Scale.

### 3.8 Spearman correlation analysis between emotion and demographic, clinical indicators

The results showed a statistically significant positive correlation between HAMA scores and the duration of T2DM (**Table 8**).

**Table 8:** Spearman correlation analysis between emotion and demographic, clinical indicators.

Variables	Age	Education	Hypertension	Drinking	Smoking	BMI	Course of disease
HAMA (*r*, *P*)	(0.094, 0.581)	(-0.111, 0.512)	(0.224, 0.183)	(0.046, 0.786)	(0.008, 0.964)	(0.079, 0.642)	(0.337, 0.045)
HAMD (*r*, *P*)	(0.17, 0.315)	(-0.265, 0.113)	(0.17, 0.313)	(-0.066,0.7)	(-0.028, 0.869)	(0.049, 0.772)	(0.251, 0.141)

HAMA, Hamilton Anxiety Scale; HAMD, Hamilton Depression Scale; BMI, body mass index.

### 3.9 Multiple linear regression of indicators

The results showed that HAMA scores had a negative impact on IIEF-5 scores, suggesting that the higher level of anxiety, the lower level of erectile function (**Table 9**).

**Table 9:** Multiple linear regression of indicators.

Variables	Unstandardized coefficients	Standardized coefficients	*Beta*	*P*
IIEF-5 scores	*B*	Standard Error		
Constant	22.375	0.824		0.000
HAMD scores	0.035	0.242	0.034	0.886
HAMA scores	-0.446	0.192	-0.549	0.024

IIEF-5, International Index of Erectile Function-5; HAMD, Hamilton Depression Scale; HAMA, Hamilton Anxiety Scale.

## 4 Discussion

In this study, a single center, cross-sectional survey was conducted on the probability of developing ED in 208 male T2DM patients. The relationship between ED and related clinical indicators, as well as risk factors, were analyzed. The findings showed that mild ED was more common in T2DM patients, while moderate and severe ED were less common. T2DMED patients were older, with higher PEDT scores and diabetic vasculopathy. In addition, the higher the age and Fins level, the more likely T2DM patients were to develop ED. HbA1C, age and education level affected the severity of ED, with higher levels of LDL, age, high risk of diabetic vasculopathy indicating more severe ED. The more severe the ED and higher PEDT scores in T2DM patients, the more severe their anxiety and depression. The more anxious T2DM patients were, the worse their sexual experienced. T2DM patients experienced an increase in anxiety levels with the duration of T2DM. As the level of anxiety increased in T2DM patients, ED became more severe. Our finding of significantly higher PEDT scores in the T2DMED group suggests a greater burden of ejaculatory complaints or dissatisfaction among these men. It should be noted that while the PEDT score indicates symptoms, it does not equate to a formal clinical diagnosis of premature ejaculation, which requires a specific cutoff and clinical evaluation. This association may reflect shared neurovascular, psychological, or medication-related etiologies common to both ED and ejaculatory dysfunction in the context of T2DM.

Previous research showed that the prevalence of ED in T2DM in China was 64.2% (42). In this study, the prevalence of ED in male T2DM patients was 67.8%. The prevalence of mild ED was 57.2%, moderate ED was 5.3%, and severe ED was 5.3%. In T2DM patients, right PWV and left PWV levels were elevated when compared with T2DM patients. PWV was considered as a biomarker of early atherosclerosis injury, and was related to atherosclerotic heart disease (43, 44). The severity of ED increased with the increase of PWV, which suggested that aortic sclerosis might affect ED (45). The population of this study was T2DM patients, and it was found that PWV in ED patients was higher than T2DM patients. It was speculated that in T2DM population, PWV might also be a marker of early atherosclerosis. However, this study had not conducted a relevant investigation on patients’ coronary heart disease, so this speculation needed further research and confirmation. ABI is the most commonly used non-invasive examination for detecting peripheral arterial diseases (46). A positive correlation between the severity of ED and left ABI was found in this study. Therefore, it was speculated that the more severe the ED, the higher the probability of peripheral arterial disease in these patients. Right ABI did not show positive correlation with ED, which might be due to insufficient sample size in this study.

In previous study, a positive correlation between ED and psychological distress was found in T2DM patients using the Symptom Checklist-90 (SCL-90) (47). In this study, there were negative correlations between IIEF-5 scores and HAMA scores, HAMD scores. There were differences in HAMA scores between T2DMED and T2DM patients. It was believed that the occurrence of ED in T2DM was related to the social and psychological status of patients. Abnormal blood lipids, especially lipoprotein levels, were associated with an increased risk of ED in T2DM (48). The results of binary logistic analysis conducted in this study suggested that TC, TG, and LDL levels did not affect the development of ED in T2DM. The results of Spearman correlation analysis showed a correlation between LDL and ED severity. Additionally, high-density lipoprotein (HDL) and lipoprotein (Lpa) were not included as risk factors for analysis in this study. Research has shown that the course of T2DM, poor blood glucose control, older age, and higher BMI were associated with the incidence of ED in T2DM patients (49). In this study, age had a positive impact on the development of ED in T2DM patients, and the severity of ED was positively correlated with age. In addition, age might predict the likelihood of ED in T2DM patients. However, this study did not find any correlation or influence between blood glucose levels, T2DM course, BMI and ED, possibly due to the older age of the subjects, low HbA1C levels, and better control of FBG levels.

Differences in depression status were found between the T2DMED and T2DM groups, and it was believed that depression status could predict the occurrence of ED (50). This study showed similar results, namely that there were negative correlations between IIEF-5 scores and HAMA scores, HAMD scores in T2DM patients. HAMA scores negatively affected IIEF-5 scores. Therefore, it was believed that social psychological states such as anxiety and depression could affect T2DMED patients, providing further clinical evidences for psychological intervention in the treatment of T2DMED. Kiskac’s study did not show a relationship between umALB and ED, and found a statistically significant relationship between HbA1C and the severity of ED (51), which was consistent with the findings in this study. However, there was controversy over whether there was a correlation between umALB, HbA1C, and ED, and more clinical studies were needed to verify it. Research had shown that higher levels of education might increase the incidence of ED in T2DM patients (42), while the results of this study showed no statistical correlation between education level and ED in T2DM patients. Further sample size expansion was needed to verify the relationship between the two factors.

Our finding that elevated fasting insulin (Fins) is an independent risk factor for ED in T2DM patients offers a novel and significant insight into the pathophysiology of T2DMED, extending beyond the traditional focus on hyperglycemia. This association underscores the pivotal role of insulin resistance and hyperinsulinemia in diabetic vasculopathy and endothelial dysfunction. Hyperinsulinemia may contribute to ED through several mechanisms: 1) It is a known promoter of vascular smooth muscle cell proliferation and atherosclerosis, thereby compromising penile arterial inflow; 2) It can stimulate sympathetic nervous system activity, leading to increased vasoconstriction; 3) It may directly impair endothelial NO synthase (eNOS) function and reduce NO production; and 4) It is closely linked to increased visceral adiposity and the secretion of pro-inflammatory adipokines, fostering a systemic inflammatory state detrimental to vascular health. This result suggests that in clinical practice, assessing insulin resistance status, perhaps through indicators like HOMA-IR, might help identify T2DM patients at a higher risk for ED even before overt vascular complications arise. It also implies that therapeutic strategies aimed at improving insulin sensitivity, such as specific pharmacological agents (e.g., GLP-1 receptor agonists, pioglitazone) and intensive lifestyle modification, may have dual benefits in improving both metabolic control and sexual function, a hypothesis that merits prospective interventional studies.

The strong correlations between ED severity, vascular parameters (PWV, ABI), and psychological scales (HAMA, HAMD) revealed in this study have profound implications for clinical practice and future research directions. Clinically, these findings advocate for a paradigm shift in the management of male T2DM patients towards integrated, multi-disciplinary care. Routine screening for ED using simple questionnaires like the IIEF-5 should be incorporated into standard diabetes care, especially for older patients and those with poor glycemic control. Positive screens should trigger a comprehensive evaluation encompassing not only glycemic and lipid profiles but also assessments of subclinical atherosclerosis (e.g., via PWV measurement) and mental health status. Management must then be equally multifaceted: optimizing glycemic control and cardiovascular risk factors forms the foundation, but it must be complemented by targeted psychosexual counseling, cognitive-behavioral therapy for anxiety/depression, and consideration of phosphodiesterase type 5 (PDE5) inhibitors, which address both the vascular and psychological components by improving erectile confidence. For future research, longitudinal studies are urgently needed to establish causality within the observed correlations. Moreover, investigating the effects of different anti-diabetic drug classes on ED incidence and progression, exploring the role of novel biomarkers like endothelial progenitor cells or specific inflammatory cytokines, and developing tailored psycho-educational interventions for this vulnerable population are critical next steps to move from understanding associations to improving patient outcomes.

More importantly, the results suggested that HbA1c, age, and high school/vocational education level were significant risk factors affecting ED severity in the multivariable model. It is noteworthy that while the bivariate Spearman correlation between HbA1c and ED severity was not statistically significant, HbA1c became a significant predictor in the ordered logistic regression analysis after adjusting for other covariates. This indicates that the relationship between glycemic control and ED severity is more clearly discernible when accounting for potential confounders such as age, BMI, and hypertension status. In addition, a notable finding of our study was the high prevalence of ED (67.8%) in a cohort with a relatively short mean T2DM duration of 2–3 years. This suggests that ED can manifest early in the course of T2DM, potentially even preceding or coinciding with the formal diagnosis. Several factors may explain this phenomenon. First, T2DM is often preceded by a prolonged period of insulin resistance and metabolic syndrome, during which subclinical endothelial dysfunction and microvascular damage accumulate. ED may thus be an early sentinel sign of this underlying vasculopathy. Second, the pathogenesis of ED in T2DM is multifactorial; factors like suboptimal glycemic control (reflected in our cohort’s mean HbA1c >8%), concurrent hypertension, dyslipidemia, and psychological distress—all present in our population—can contribute to ED independently of, or synergistically with, diabetes duration. Third, our cohort’s mean age was mid-40s, an age where vasculogenic factors begin to significantly contribute to ED. The high prevalence highlights the importance of routine sexual health screening in all men with T2DM, regardless of disease duration.

The high prevalence of ED (67.8%) in our cohort means that the odds ratios (ORs) reported from our binary logistic regression analyses likely overestimate the strength of associations compared to risk ratios or prevalence ratios. The highly imbalanced distribution of ED severity (predominantly mild) may limit the precision of estimates for factors specifically associated with moderate-to-severe ED in our ordered logistic regression model. Future cross-sectional studies with a high outcome prevalence would benefit from employing analytical methods, such as modified Poisson regression, that generate prevalence ratios for more directly interpretable measures of association. Additionally, the duration of T2DM was calculated from the time of formal diagnosis. The potential long, undiagnosed preclinical phase of diabetes means the true duration of metabolic disturbance may be underestimated, which could influence the association between disease duration and complications like ED. Furthermore, this study did not assess hormonal parameters such as testosterone or sex hormone-binding globulin levels, which are known to influence both metabolic health and sexual function. Future studies incorporating these biomarkers would provide a more comprehensive understanding of the endocrine pathways involved in T2DM-related ED.

However, several limitations should be considered in this study. First, all participants were of Han ethnicity, limiting the generalizability of our findings to other ethnic groups. Second, we did not collect detailed data on lifestyle factors, which are known to influence both metabolic health and sexual function. Third, while we recorded major medication classes, a more granular analysis of specific antidiabetic, antihypertensive, and psychotropic medications and their doses was not performed. Fourth, although we assessed vascular parameters (PWV, ABI), we did not systematically document the presence or severity of established macrovascular or microvascular complications. Fifth, we did not measure several biomarkers implicated in endothelial dysfunction and ED.

## 5 Conclusion

In this study, the findings suggested that T2DM patients had a high probability of ED. The incidence rate and severity of ED in T2DM patients were related to age, glycosylated hemoglobin, vascular disease, neuropathy and other factors. Patients with T2DM had social, emotional, and cognitive barriers when developing ED, which explained the psychological reasons for ED, namely that T2DM patients with anxiety or depression were prone to developing ED. Therefore, it was suggested that clinical attention should be paid to the erectile status of male patients with T2DM, and early screening of ED population, early intervention and treatment should be carried out as far as possible.

## Data Availability

The raw data supporting the conclusions of this article will be made available by the authors, without undue reservation.
